# The Physiological Standardisation of Drugs

**Published:** 1899-09-23

**Authors:** 


### THE PHYSIOLOGICAL STANDARDISATION OF DRUGS.

A suggestion is likely to be brought up before the committee which will meet next year for the decennial revision of the American Pharmacopœia which will tend to place the ever interesting problem of the strength of drugs upon a more satisfactory basis than it occupies at present. Not in regard to all drugs do we as yet possess even a chemical standard of strength, and there can be little doubt that one at least of the reasons by which prescribers are led to use the pure alkaloids rather than the crude drugs is that in the one case the therapeutic energy of the drug is known, whereas in the other it is a sadly varying quantity. But there are many drugs in regard to which chemistry and even the most exact analysis speak with but uncertain voice in regard to physiological activity, and the question is now raised whether it would not be desirable in standardising drugs to adopt the principles with which we have now for some time been familiar in regard to the standardising of the toxins and antitoxins used in the pathological laboratory. As is well known, some enterprising firms of pharmaceutists have already adopted a similar plan, and guarantee that a given dose of certain of the drugs they issue will produce a certain physiological effect, and, indeed, it is only in this way that it is possible to estimate the dosage of some of the more recent preparations of snake poison and the like. In view, then, of the fact that it is in reference to some of the strongest of our drugs—drugs the exact dosage of which is of the utmost importance—that the greatest difficulty exists in obtaining exact and useful results from chemical analysis, it does not seem unreasonable to suggest that instead of drugs being so standardised that a given quantity shall contain a given weight of some one alkaloid (which may or may not be the most active ingredient), the drugs shall be issued of such a strength that a given quantity shall produce a given physiological effect. It is a suggestion which is worthy of careful consideration; albeit we are afraid that if accepted the price of guinea-pigs will go up.

